# Possible Mechanisms of Fullerene C_**60**_ Antioxidant Action

**DOI:** 10.1155/2013/821498

**Published:** 2013-10-08

**Authors:** V. A. Chistyakov, Yu. O. Smirnova, E. V. Prazdnova, A. V. Soldatov

**Affiliations:** ^1^Research Institute of Biology, Southern Federal University, Rostov-on-Don 344090, Russia; ^2^Research Center for Nanoscale Structure of Matter, Southern Federal University, Rostov-on-Don 344090, Russia; ^3^Department of Physics, Purdue University, West Lafayette, IN 47907, USA

## Abstract

Novel mechanism of antioxidant activity of buckminsterfullerene C_60_ based on protons absorbing and mild uncoupling of mitochondrial respiration and phosphorylation was postulated. In the present study we confirm this hypothesis using computer modeling based on Density Functional Theory. Fullerene's geroprotective activity is sufficiently higher than those of the most powerful reactive oxygen species scavengers. We propose here that C_60_ has an ability to acquire positive charge by absorbing inside several protons and this complex could penetrate into mitochondria. Such a process allows for mild uncoupling of respiration and phosphorylation. This, in turn, leads to the decrease in ROS production.

## 1. Introduction

Reactive oxygen species (ROS) are able to cause oxidative damage to DNA, lipids, and proteins and are known to be the key regulators of cellular signaling. In spite of the criticism from a number of researchers [1] free-radical theory occupies a pivotal position in modern biological concepts of aging [2]. The ability to retard senescence is typical for many antioxidants [3–5]. Well-known ability of fresh vegetables, fruits, red wines, and spices to stimulate longevity is largely determined by the existence of compounds such as deprotonated xanthones [6], carotenoids [7], anthocyanins and pyranoanthocyanins [8], and flavonoids and terpenoids [9]. These compounds exhibit a broad spectrum of oxyradical quenching activity based on reactions of single electron transfer, hydrogen atom transfer, sequential electron proton transfer, proton coupled electron transfer, radical adduct formation, and iron chelation [5, 9–13].

In the recent study Baati et al. [14] showed that the oral administration of the fullerene C_60_ suspension in olive oil retards senescence of rats. Herewith, median and maximum life span increase approximately twice. Moreover, it was shown that rats treated with fullerene C_60_ demonstrated high resistance to carbon tetrachloride. Toxicity of this substance is mediated by ROS generation [15]. According to this fact and results of biochemical tests fullerene C_60_ was proposed to be of high antioxidant activity *in vivo*. Due to the free-radical theory of aging, highly active antioxidant activity can be the basis for unique antiaging (geroprotective) properties.

Fullerene C_60_ is known to be able to inactivate hydroxyl radicals by attaching to double bonds [16]. However, this mechanism cannot explain sufficient (near two times) increase in lifespan of rats. Such kind of antioxidative activity is also attributed to natural phenolic antioxidants that do not possess high senescence retarding activity [17]. We propose that there is an additional mechanism involved in fullerene anti-aging activity. Respiratory chain located in the inner mitochondrial membrane is the main source of superoxide anion radicals, which lead to a cascade of other toxic ROS. In this connection mitochondrial-targeted antioxidants like lipophilic cations (Skulachev ions) with antioxidant load [18] are the most effective antiaging agents (geroprotectors) among synthetic compounds.

Accumulation of Skulachev ions in the mitochondria is based on the transmembrane potential difference generated as a result of electron transport chain activity. The outer side of inner membrane of mitochondria has positive charge and the inner side has negative charge. So, lipophilic cations are concentrating in mitochondria via electric field forces [18]. The lipophilic properties of fullerene C_60_ are well known [19]. In addition, Wong-Ekkabut et al. showed using molecular dynamics simulations [20] that C_60_ fullerene is capable of penetrating into membrane and accumulates in the middle of lipid bilayer. However, the simulation does not consider the possible presence of fullerene and/or membrane charge. We suppose that fullerene is capable of absorbing protons and obtaining positive charge, which allows it to be delivered into the mitochondria. Thus, superoxide anion-radical generation is decreased by mild uncoupling of respiration and phosphorylation [21]. In the present study we perform theoretical analysis of the fullerene C_60_ ability to acquire positive charge and to absorb protons to prove that the proposed mechanism indeed may take place.

## 2. Methods

All the computer simulations were performed within the framework of Density Functional Theory (DFT) for solving Schrödinger equation [22], which has been used for the investigation of antioxidants previously [23]. In the present work, DFT implemented in ADF 2012 code was used [24]. Initially from one up to seven protons were placed outside the fullerene and then the most probable atomic configuration was found by minimizing the total energy of the system during the process of geometry optimization, that is, finding a stable configuration of the system that corresponds to the minimum of total energy. For the exchange-correlation part of molecule potential General Gradient Approximation (GGA) was used in both GGA-BLYP [25] and GGA-BLYP-D3 [25, 26] forms, but all final results were obtained using GGA-BLYP potential. Basis sets are DZ (double-*ζ*) within the calculations including water molecules around C_60_ and TZP (triple-*ζ*) within the calculations without taking into account the water molecules around “C_60_ plus-protons” system.

## 3. Results

**Figure 1:**
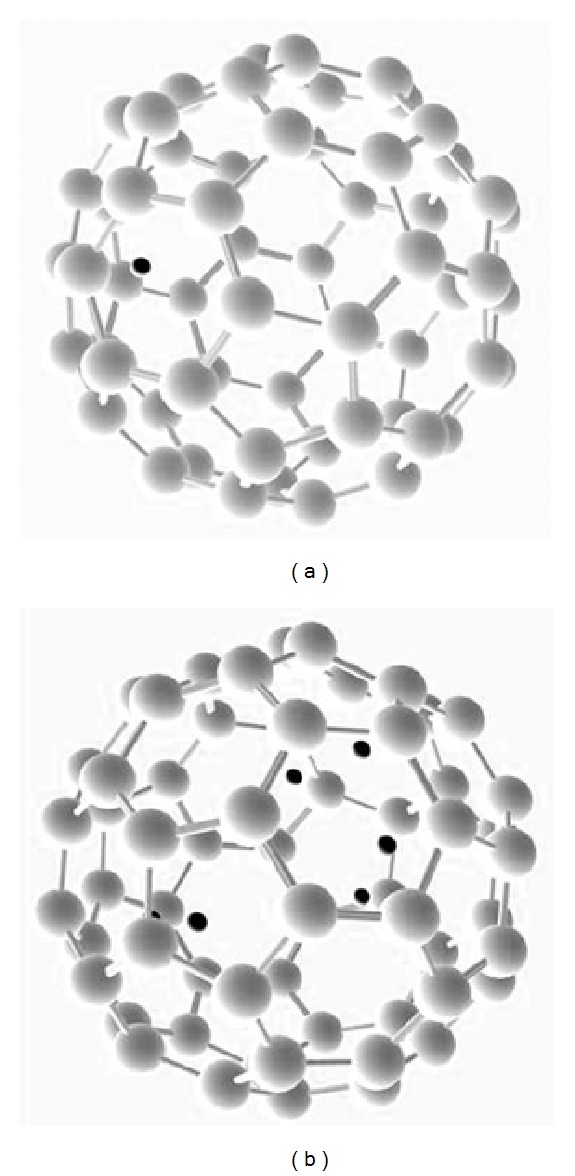
The results of DFT geometry optimization for one (a) and six (b) protons and fullerene. Initially protons were placed outside the fullerene and then the configuration that has the minimum value of total energy was found as a result of DFT geometry optimization. As a result, all protons appeared to be inside the fullerene. For the simulation, GGA-BLYP exchange-correlation potential was used. Carbon atoms are shown in grey and protons are shown in black.

At first step an interaction between single proton and fullerene was simulated. The proton was placed outside the C_60_ above one of the pentagons at the distance about 1 Å from the pentagon plane. As a result, the proton transfers into the fullerene and finally appeared to be inside the fullerene at a distance about 1.1 Å from the nearest carbon atom (Figure 1(a)). Next, more protons were added to this system; some of them were initially placed above pentagons, but most were placed above hexagons. The first two protons were placed at maximum possible distance from each other. All others were equally distributed around the fullerene. In all cases protons were “absorbed” by the fullerene, and it was so until the seventh proton was added to the system—it repulsed from the fullerene. So, the maximum amount of protons inside the fullerene consists of six protons (Figure 1(b)).

**Figure 2:**
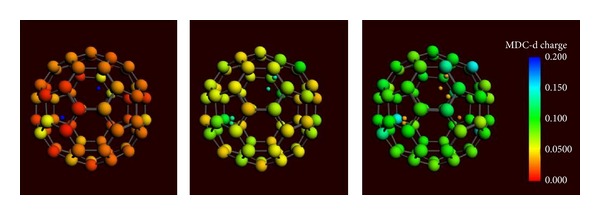
The distribution of charge for two, four, and six protons inside the fullerene. The charge of fullerene with two protons inside is about zero (red color) while fullerenes that have four or six protons inside obtain positive charge (green and blue color). Protons lose their positive charge starting from positive charge (blue color) to almost zero (orange color).

It is crucial to know the distribution of charge over C_60_ for each configuration of protons.  Figure 2 shows the distribution for two, four, and six protons inside the fullerene. It can be seen that when there are two protons inside the surface of the fullerene has almost no charge. When four to six protons are added the fullerene surface obtains positive charge.

Table 1 provides information about binding energies and VDD charges [27] for each proton added to the system. Both charges on protons and relatively big C-H distances allow us to suppose that protons interact with fullerene according to donor-acceptor mechanism and do not form strong chemical bonds.

It is important to know whether the presence of other molecules near fullerene will impact the ability of protons to penetrate into fullerene or not. For this purpose we performed a simulation involving water molecules which are the most common in organisms. Though it is known that in the presence of both protons and water hydronium ions will appear, water molecules can be chosen. An exchange of protons between hydronium ions takes place in such environment, so for some small period of time protons are free.

The simulation was carried out for a fullerene with single proton placed above a pentagon and 47 water molecules randomly distributed around the fullerene. It was shown that solvent molecules do not influence the capability of a fullerene to absorb the proton.

## 4. Discussion

According to our model fullerene C_60_ accumulating in mitochondria provides high radical scavenging activity in this subcellular compartment, called by Skulachev the “dirtiest place in the cell” [28]. Another effective antioxidant mechanism is based on mild uncoupling of respiration and phosphorylation. Respiratory chain obtains electrons from NADH and succinate. They are used for harmless four-electron reduction of oxygen. But the transfer of one or two electrons could produce the radicals that are dangerous to cells (such as superoxide or peroxide anions).

The specific feature attributable to the generation of ROS by mitochondria is related to the fact that the higher is the membrane potential (the larger is the difference in the concentration of protons inside and outside the mitochondria), the higher is the level of the superoxide anion production. As it was shown [29], there is steep dependence of mitochondrial superoxide-anion-radical generation on transmembrane potential (Δ*ψ*). Even a small (10–15%) decline of Δ*ψ* resulted in tenfold lowering of ROS production rate.

Therefore, the so-called mild uncouplers of oxidative phosphorylation are the substances which can move some of the protons inside the mitochondria and can possess an excellent oxygen-protective effect, although they are not antioxidants in terms of chemistry [19].

DFT simulations allowed us to propose the following mechanism. C_60_ fullerene molecules enter the space between inner and outer membranes of mitochondria, where the excess of protons has been formed by diffusion. In this compartment fullerenes are loaded with protons and acquire positive charge distributed over their surface. Such “charge-loaded” particles can be transferred through the inner membrane of the mitochondria due to the potential difference generated by the inner membrane, using electrochemical mechanism described in detail by Skulachev et al. [18, 24]. In this case the transmembrane potential is reduced, which in turn significantly reduces the intensity of superoxide anion-radical production.

## 5. Conclusion

The proposed ability of C_60_ fullerenes to acquire positive charge allows ascribing them to the mitochondrial-targeted compounds. The key role of mitochondria in the cellular regulation makes such “charge-loaded” fullerenes be of great interest along the route for novel classes of drugs development.

## Figures and Tables

**Table 1:** Binding energies and VDD charges for different amounts of protons added to fullerene.

Number of protons	Binding energy values, eV	The Voronoi Deformation Density (VDD)
1	18.01	0.350

2	22.34	0.345 0.333

3	22.97	0.334 0.314 0.312

4	28.69	0.319 0.308 0.304 0.268

5	28.71	0.281 0.299 0.302 0.278 0.328
